# The application of gradually expanding pedicle technology in the placement of screws in thinner pedicle of patients with idiopathic scoliosis

**DOI:** 10.3389/fsurg.2024.1388534

**Published:** 2024-07-18

**Authors:** Fei Chen, Jianyi Li, Tao Li, Yongming Xi

**Affiliations:** ^1^ Department of Spinal Surgery, The Affiliated Hospital of Qingdao University, Qingdao, Shandong, China; ^2^ Department of Spine Surgery, Shandong Provincial Hospital Affiliated to Shandong First Medical University, Jinan, Shandong, China

**Keywords:** scoliosis, pedicle screw, pedicle expansion, spine, surgery

## Abstract

**Objective:**

To propose a technique of gradual expansion of pedicle diameter to place screws on the thinner pedicle that is difficult to place screws in scoliosis orthopedic surgery, in order to place thicker pedicle screws in the premise of good safety to achieve good stability and orthopaedic effect.

**Methods:**

The authors reviewed that 36 patients with Adolescent idiopathic scoliosis (AIS) (20 females, 16 males) in our department from June 2020 to March 2024 underwent posterior spinal correction and internal fixation were enrolled in the present study. 194 pedicles had narrower diameter ranging from 0 mm to 4.5 mm. After analysis, 155 pedicles (internal diameter: 2.5 mm–4.5 mm) were treated with gradually expanding pedicles technology. The angle between the inserted screws and the upper endplate of the vertebral body was measured 1 week after operation and 3 months after operation to evaluate the placement of the screws after pedicle expansion.

**Results:**

All operations were completed without dura mater rupture, nerve root injury, infection and poor incision healing. There was no screw breaking or screw pull-out in DR film at 3 months after operation compared with 1 week after operation. There was no significant difference in the angle between screw and upper endplate measured by the three observers at 3 months and 1 week after operation (Wilcoxon's signed rank test, *p* > 0.05). In the measurement at 1 week or 3 months after operation, there was no statistically significant difference among the three observers. There is a good consistency between the observers (Cronbach's alpha > 0.80).

**Conclusion:**

In AIS patients, the thinner pedicle with a diameter range of 2.5 mm to 4.5 mm can be safely inserted with relatively thicker pedicle screws after gradually expanding pedicle technology.

## 1 Introduction

Pedicle screw-rod system internal fixation and orthopedic surgery has become the most effective method for the treatment of severe Adolescent idiopathic scoliosis (AIS), which has been widely recognized all over the world (1). The stability of screw placed in pedicle is one of the most important guarantees for the success of orthopedic surgery for spinal deformity. In previous studies, it has been found that there are thinner pedicles in AIS patients, and the finer pedicles are mostly located on the concave side, and the diameters of 62% of the T3-T9 concave side pedicle are less than 4 mm in patients with AIS (2–4), which makes it difficult to place pedicle screws, and some are even too thin to place pedicle screws. When placing screws in thinner pedicles, there is a greater risk of screws penetrating the pedicle walls. Postoperative CT examinations by Z. F. Zhang in 21 patients with scoliosis and 47 patients with non-scoliosis showed that the incidence of screws penetrating the pedicle wall is 8% in patients with scoliosis and 1% in patients with non-scoliosis (5). It is speculated that this difference is attributed to more thinner pedicles in scoliosis patients. When the pedicle is too thin to place normal screws, the surgeons would usually place pedicle external screws or thinner screws, which are far less stable than transpedicular screws or relatively thick screws, which will increase the risk of instability of internal fixation and screw pullout.

In view of the fact that it is difficult to place normal screws in thinner pedicles, we make use of the characteristics of high expansibility of pedicles in children (6), using gradually expanding pedicle technology (GEPT) in scoliosis correction surgery, in order to place thicker pedicle screws in the premise of good safety to achieve good stability and orthopaedic effect.

Some studies have shown that the pedicle of children has higher expansibility, it is reported that the diameter of pedicle screw can be 115% of the transverse diameter of pedicle to be fixed, and the internal diameter of pedicle can be expanded by 74% at most (6, 7). Accordingly, we define the value of thinner pedicle diameter of pedicle which can use gradually expanding pedicles technology (GEPT) as 2.5 mm–4.5 mm. Subsequently, in scoliosis correction surgery, screw placement is performed on such thinner pedicles that are difficult to place screws, in order to place thicker pedicle screws under the premise of good safety to achieve good stability and orthopedic effect.

## 2 Material and methods

### 2.1 Patients and pedicles

36 patients with AIS (20 females, 16 males) in our department from June 2020 to March 2024 underwent posterior spinal correction and internal fixation were enrolled in the present study. The average age is 12.59 years old (range: 4–15). 194 pedicles had narrower diameter ranging from 0 mm to 4.5 mm. After analysis, 155 pedicles (internal diameter: 2.5 mm–4.5 mm) were treated with gradually expanding pedicles technology, and neuroelectrophysiological monitoring was used in all cases. The inclusion criteria are as follows: (1) scoliosis which was confirmed by DR in the anterior and lateral position of the whole spine. (2) For patients who need surgical treatment, further CT examination and measurement of pedicle diameter were performed, and the pedicle with diameter larger than 2.5 mm and less than 4.5 mm were selected. The exclusion criteria are as follows: (1) There was no cancellous bone in the cortical junction of the pedicle. (2) CT measurement of pedicle diameter is less than 2.5 mm, which may lead to a high risk of rupture. (3) CT measurement of pedicle diameter is more than 4.5 mm, 5.0 mm screws can be inserted with pedicle toughness. (5) Patients complicated with other diseases affecting bone.

### 2.2 Method of measuring the internal diameter of pedicle

**Figure 1:**
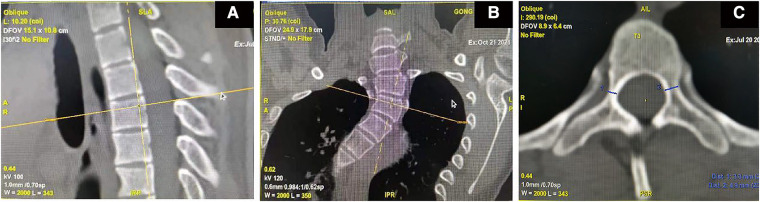
Coronal and sagittal images are obtained from axial images obtained by CT plain scan. Select the vertebral body to be measured on the sagittal image so that the longitudinal positioning table is located at the posterior edge of the vertebral body (**A**) In the coronal position, the transverse positioning mark is located at the upper edge of the vertebral body to determine the need to measure the vertebral body. (**B**) Adjust the vertebral body level on the axial position, follow the moving observation to find the narrowest position of the pedicle on the coronal position, measure the width of the pedicle at the narrowest position of the pedicle on the axial position, and obtain the data of the diameter of the pedicle for 2D, as shown in T3, the pedicle is 3.3 mm on the right and 4.9 mm on the left (**C**).

**Figure 2:**
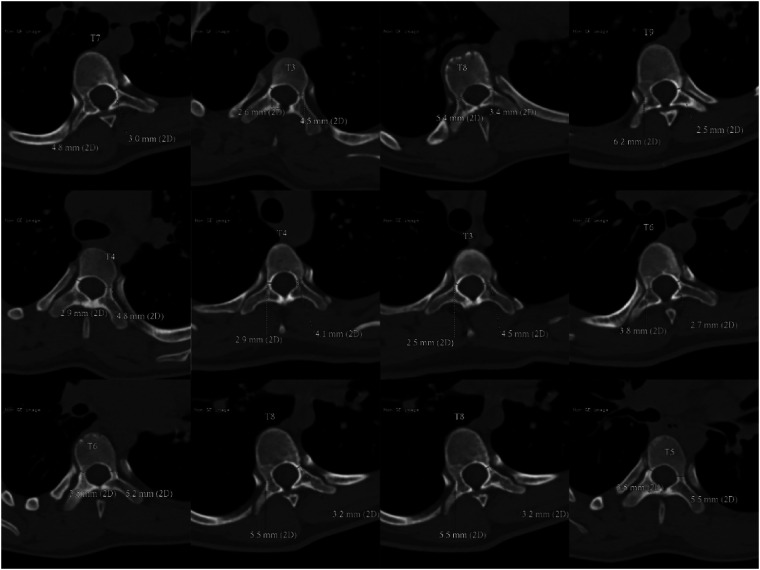
Measurement results of thinner pedicle of partial composite included in the criteria.

**Figure 3:**
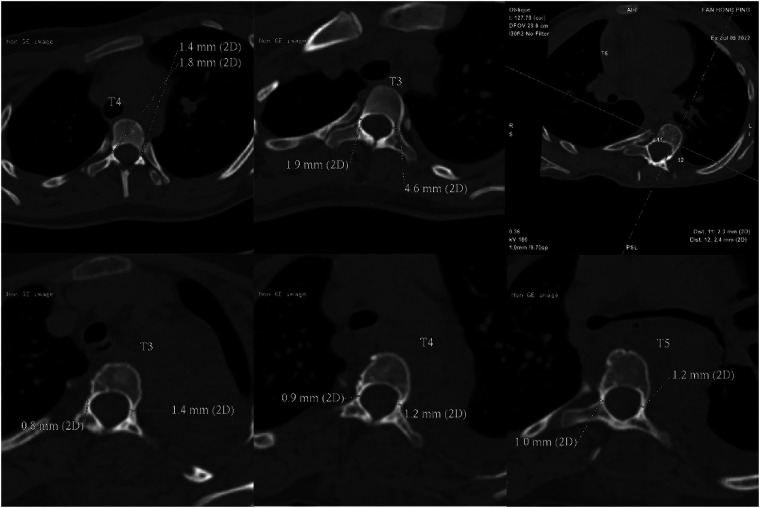
Pedicle too small in diameter to screw. Only cortical bone connection without cancellous bone pedicle that internal diameter was less than 2.5 mm.

The patient was scanned with 64 row CT scanner (Erlangen Siemens Medical Products, Germany) under calm breathing state. Parameters: layer thickness 5 ram, scanning time about 10 s, pitch 1.375:1, 120 kV, 150 mA; FOV:36 cm; The matrix is 512 × 512, and the original image data is reconstructed with a standard algorithm, with a layer thickness of 0.625 turn. In the post-processing, Siemens syngo workstation was used to obtain the coronal, sagittal and transverse images of the vertebral body under the multiplanar reconstruction technology, and the internal diameter of the pedicle on both sides was measured according to the corresponding position in syngo. As shown in Figure 1, take T3 vertebral body as an example. All 36 patients performed this measurement method, and selected the pedicle that is difficult to place the screws in a smaller size, and prepared it for gradual pedicle diameter expansion (Figure 2). It was ruled out that only the cortical bone was connected without cancellous bone pedicle (Figure 3).

### 2.3 Gradual expansion of pedicle surgery

**Figure 4:**
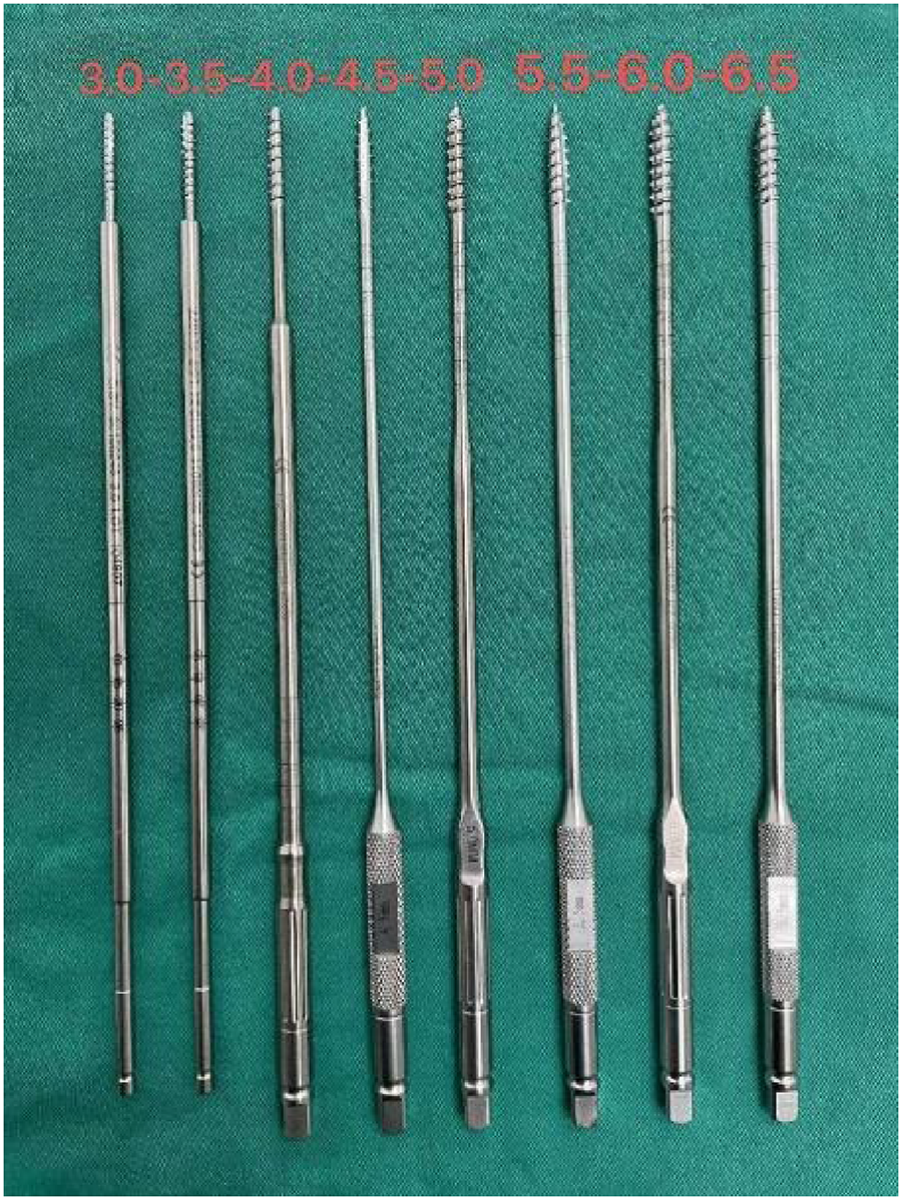
Wiretapping. The “wiretapping” device with diameter 3.0 mm–6.5 mm is used as the gradual expansion of pedicle surgery instrument, which is made of medical stainless steel and is composed of screw head end, rod and shank. The screw ends are blunt and smooth compared with conventional screws, and the rod parts are longer and harder.

**Figure 5:**
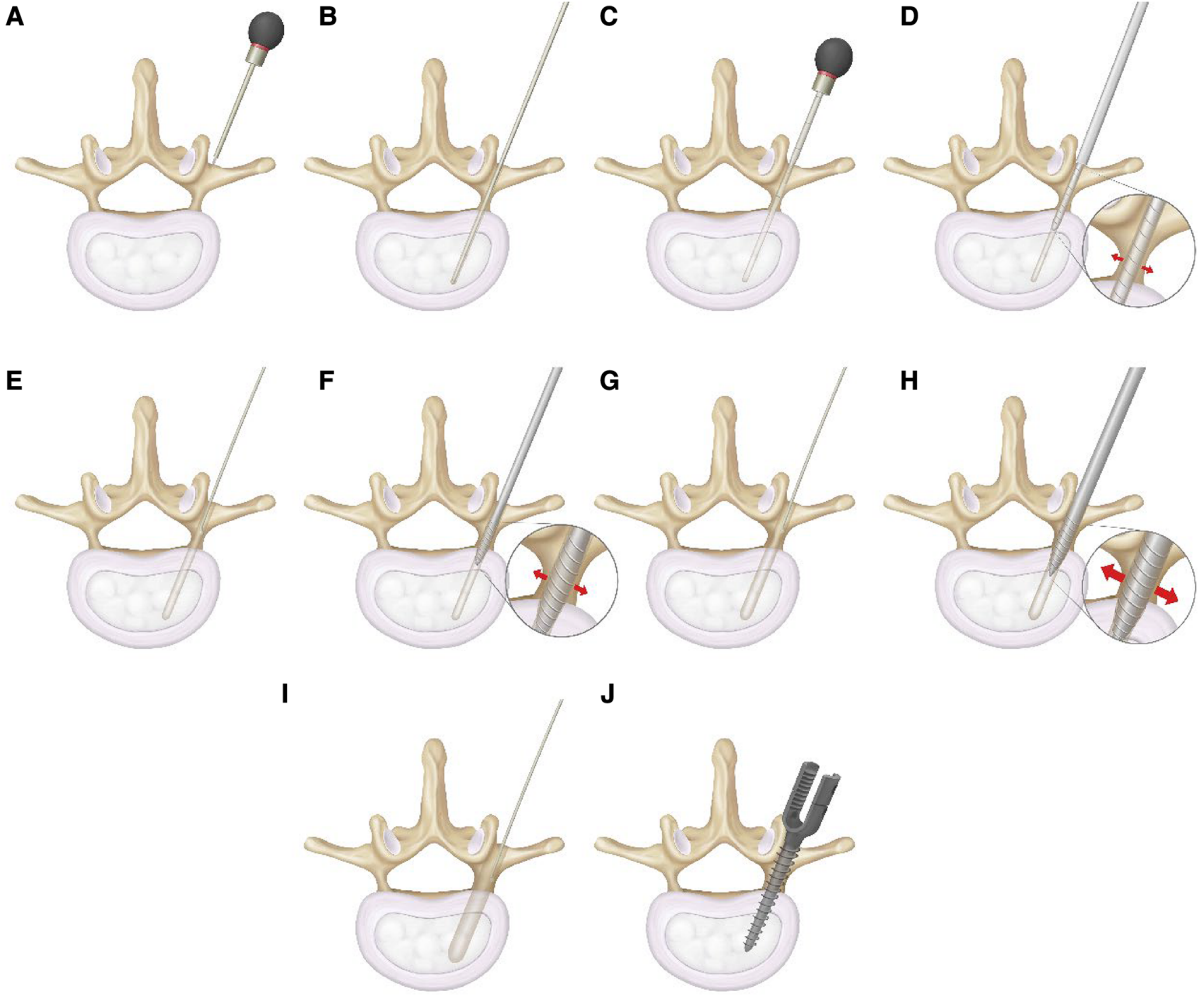
Pedicle expansion method. At the entry point of the pedicle, the opener is punctured, and at the same time, the fine cone probe is used to determine the screw path (**A**–**C**). The screw path spirally enters the cancellous mass of the pedicle and reaches the vertebral body by the probe. After withdrawing the expansion instrument, the inner wall of the pedicle is detected with a soft probe (**D**–**E**), the internal diameter of the expander is increased step by step, and the inner wall of the pedicle is continuously detected by soft probe. Stop dilatation when the pedicle diameter is larger than that of the preoperative pedicle or when the inner wall of the pedicle is damaged by a soft probe, and then insert the pedicle screw (**F**–**J**).

GEPT uses the “wiretapping” device of diameter 3.0 mm–6.5 mm as a step-by-step expansion instrument, which is composed of screw head end, rod and shank. The thread on both sides of the screw head end is blunt and smooth compared to the conventional screw, and the rod part is longer and stiff (Figure 4). All operations were performed by senior doctors. After the exposure, the pedicle screw was punctured smoothly, and the spherical probe technique (8) was used to determine the nail path (Figures 5A–C). First of all, the screw channel determined by the probe is spirally planted into the spongy of the pedicle and into the vertebral body, and the inner wall of the pedicle is detected with a soft probe after withdrawing the dilatation instrument. then gradually increase the internal diameter of the expansion instrument and constantly use the soft probe to detect the inner wall of the pedicle, so as to stop the expansion when the instrument type is larger than the preoperative pedicle diameter or when the pedicle inner wall is damaged. Pedicle screws were then placed (Figures 5D–J).

### 2.4 Assessment of the clinical outcomes

**Figure 6:**
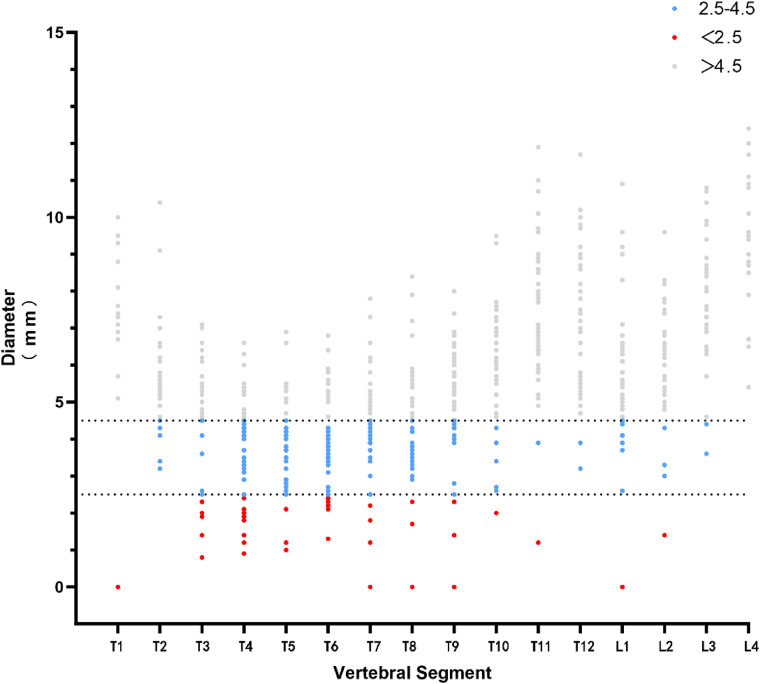
Distribution of pedicle diameter and screw placement protocol. The blue part is the pedicle between 2.5–4.5 mm, using GEPT; the red part is the pedicle <2.5 mm, without using conventional placement and GEPT; the gray part is the pedicle >4.5 mm, using conventional placement techniques.

**Figure 7:**
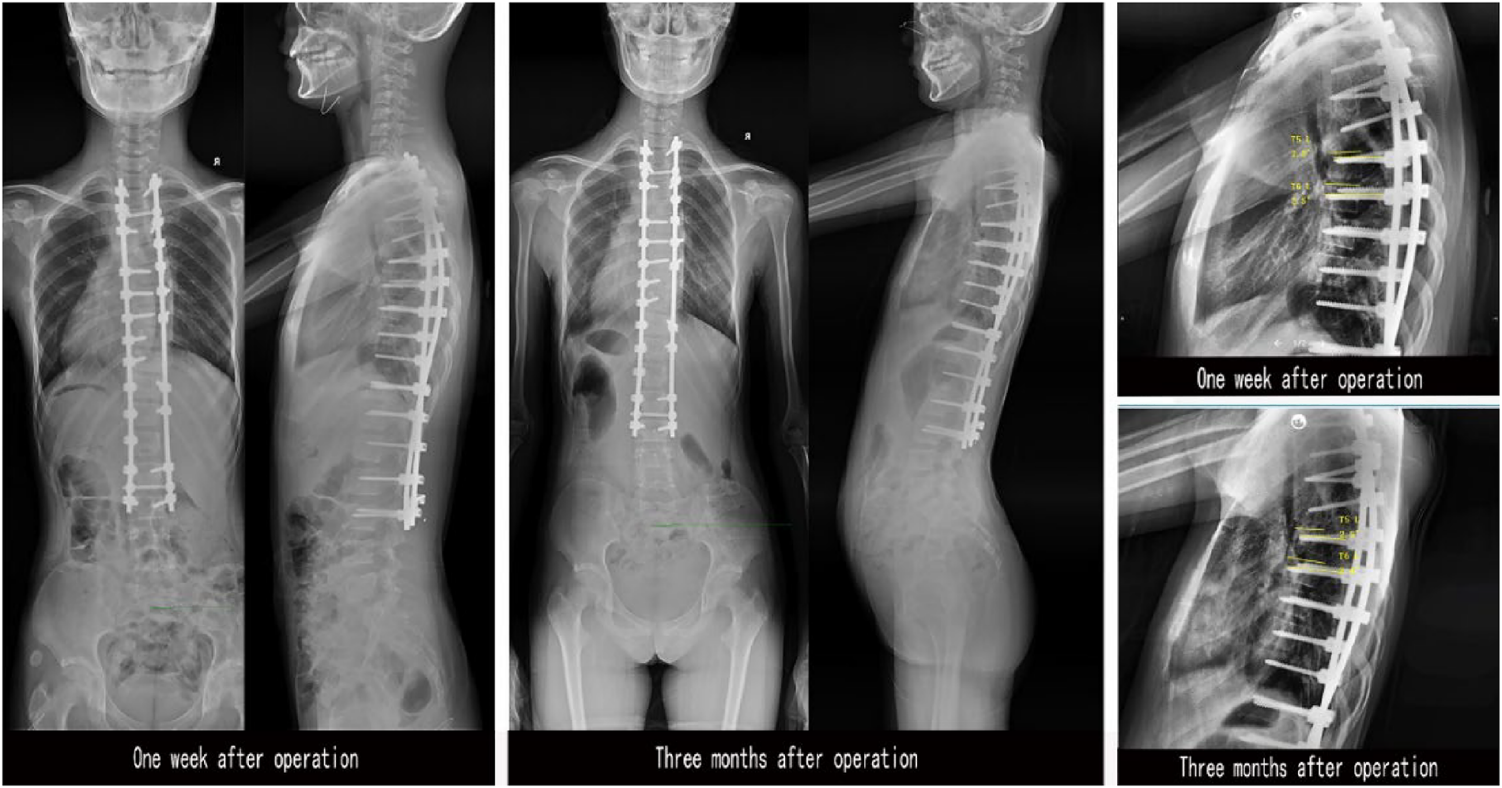
DR examination at 1 week and 3 months after surgery. The anterior and lateral DR for one week after operation, was compared with the anterior and lateral DR for 3 months after operation. The left and right screws on the lateral DR were determined according to the screw direction of the anterior DR, and the angle between the upper endplate and the screw was measured on the lateral DR.

After surgery, when the patient's lower limbs are moving and feeling well, a full spine standing position and a lateral position DR are taken at 1 week after the removal of the drainage tube and 3 months after surgery for follow-up. The orientation of the pedicle screw tip is assessed on the posterior-anterior x-ray, and the lateral distribution of the pedicle screws is clarified. The angle between the screw inserted after the gradual expansion of the pedicle and the endplate on the vertebral body is measured and compared to analyze the stability of the screw (Figure 6). Because of the radiation factors, we did not perform postoperative CT scan for all patients, but only 2 patients who were suspected of screw fracture due to accidental fall out of bed were scanned by CT, and the postoperative pedicle diameter was measured (Figure 7). All postoperative imaging measurements were performed by two experienced spinal surgeons and one experienced radiologist. Then statistical analysis was carried out.

### 2.5 Statistical analysis

Use descriptive statistics to describe continuous variables (average, standard deviation, minimum, median, maximum). The Cronbach *α* coefficient was used to test the interobserver consistency of continuous variables. Wilcoxon signed rank test is used to test the correlation between two related and non-normal distribution related continuous variables. In all analyses, *p* < 0.05 indicates statistical significance. Statistical analysis using SPSS25.0 software.

## 3 Result

**Figure 8:**
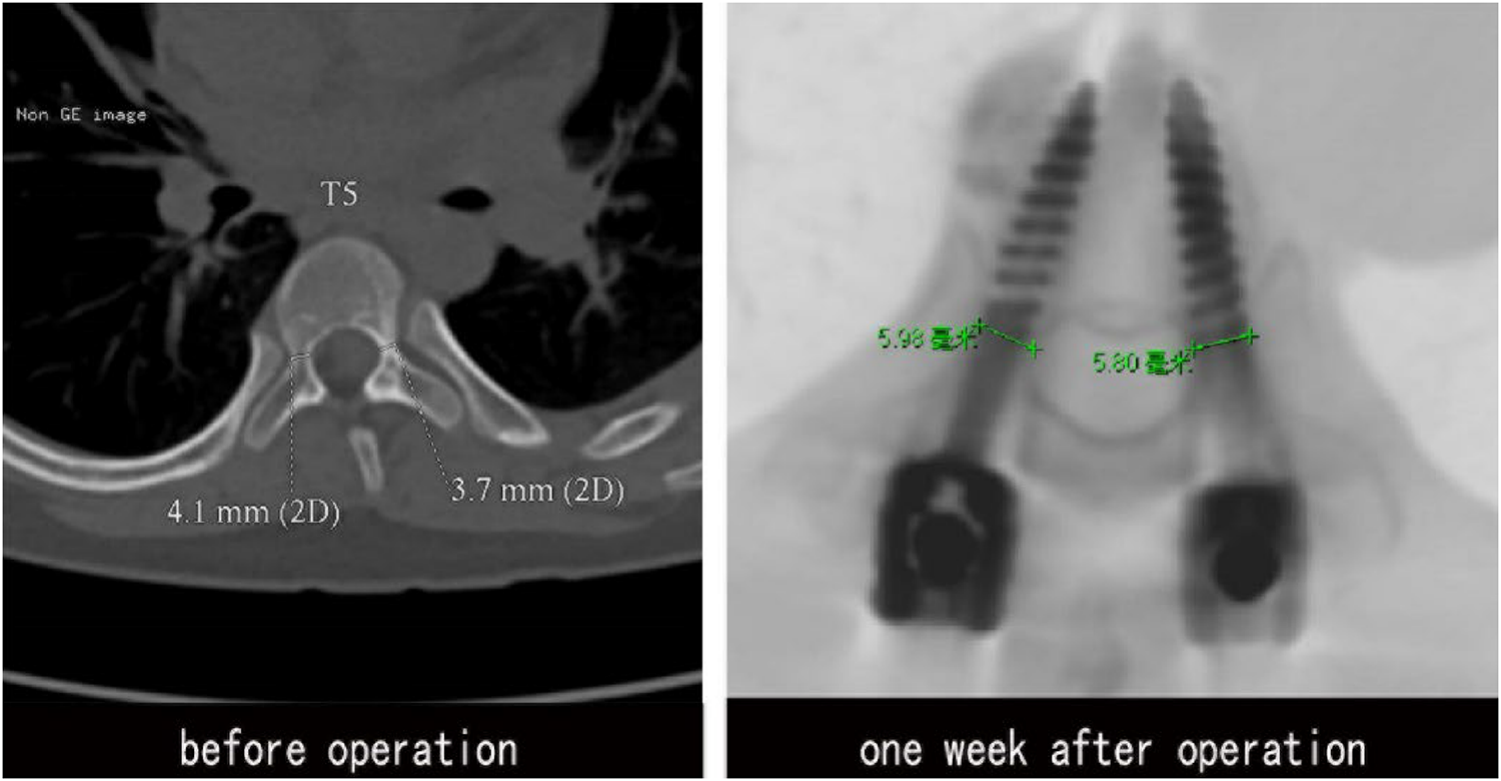
CT examination at 1 week. Before operation, the internal diameter of pedicle was 4.1 mm (**R**), 3.7 mm (**L**). GEPT was performed on the left pedicle, and 5.0 mm pedicle screws were placed on both sides. The internal diameter of pedicle was measured by CT after operation: 5.98 mm (**R**), 5.80 mm (**L**). No bone cortical phenomena of pedicle rupture and screw penetration into the medial and lateral pedicle and the anterior wall of vertebral body were found.

The pedicle measurements were recorded (Table 1), and the screw placement scheme was determined according to the diameter. The pedicle smaller than 2.5 mm did not place the screw, the pedicle between 2.5–4.5 mm used GEPT technique, and the pedicle larger than 4.5 mm used conventional screw placement (Figure 8).

**Table 1:** Measurement of pedicle diameter.

Vertebral segment	*n*	Diameter (mm)
T1	14	7.1 ± 2.5
T2	30	5.6 ± 1.5
T3	38	4.4 ± 1.6
T4	42	3.8 ± 1.4
T5	42	4.0 ± 1.3
T6	42	4.4 ± 1.6
T7	42	5.1 ± 1.5
T8	44	4.6 ± 1.6
T9	44	5.1 ± 1.5
T10	44	5.9 ± 1.6
T11	44	7.4 ± 2.0
T12	42	7.1 ± 1.9
L1	42	5.7 ± 1.9
L2	38	6.1 ± 1.6
L3	32	7.8 ± 1.8
L4	22	9.2 ± 1.8

All operations were completed without dura mater rupture, nerve root injury, infection and poor incision healing. The number of 194 pedicle screws placed horizontally per vertebral body is as follows: T2 = 5, T3 = 11, T4 = 21, T5 = 27, T6 = 21, T7 = 17, T8 = 44, T9 = 11, T10 = 6, T11 = 1, T12 = 2, L1 = 9, L2 = 2, L3 = 4, L4 = 2. A total of 83 vertebral pedicles in 27 patients were reviewed according to the doctor's advice, and DR Data were obtained 3 months and 1 week after surgery. There was no screw breaking or screw pull-out in DR film at 3 months after operation compared with 1 week after operation. There was no significant difference in the angle between screw and upper endplate measured by the three observers at 3 months and 1 week after operation (Wilcoxon's signed rank test, *p* > 0.05; Table 2). In the measurement at 1 week or 3 months after operation, there was no statistically significant difference among the three observers. There is a good consistency between the observers (Cronbach's alpha > 0.80; Table 3).

**Table 2:** Measurement of the angle between the screw and the upper endplate at 3 months and 1 week after surgery by three different observers (interobserver).

	1 week after operation (°)	3 months after operation (°)	*P*
	Average ± standard deviation Med (min - max)	Average ± standard deviation Med (min - max)	
Observer 1	2.61 ± 0.25 2.6 (2.0–3.3)	2.60 ± 0.25 2.6 (2.1–3.2)	0.440
Observer 2	2.62 ± 0.25 2.6 (2.1–3.2)	2.62 ± 0.25 2.6 (2.1–3.2)	0.365
Observer 3	2.63 ± 0.23 2.6 (2.1–3.2)	2.63 ± 0.24 2.6 (2.1–3.3)	0.655

There were no significant differences between 1 week after operation and 3 months after operation measurements (Wilcoxon's signed rank test, *p* > 0.05).

**Table 3:** Comparison of measurements made by three different observers (interobserver).

	Observer 1	Observer 2	Observer 3	*P*
	Average ± standard deviation Med (min - max)	Average ± standard deviation Med (min - max)	Average ± standard deviation Med (min - max)	
1 week after operation	2.61 ± 0.25 2.6 (2.0–3.3)	2.62 ± 0.25 2.6 (2.1–3.2)	2.63 ± 0.23 2.6 (2.1–3.2)	0.945
3 months after operation	2.60 ± 0.25 2.6 (2.1–3.2)	2.62 ± 0.25 2.6 (2.1–3.2)	2.63 ± 0.24 2.6 (2.1–3.3)	0.888

There was good concordance among the observers (Cronbach's alpha, >0.80).

2 patients who were suspected of screw fracture due to accidental fall out of bed were scanned by CT, and the postoperative pedicle diameter was measured (Figure 7), Before operation, the internal diameter of pedicle was 4.10 mm (R), 3.70 mm (L). GEPT was performed on the left pedicle, and 5.0 mm pedicle screws were placed on both sides. The internal diameter of pedicle was measured by CT after operation: 5.98 mm (R), 5.80 mm (L). Compared with before, it expanded by 45.9% and 56.8% respectively.

## 4 Discussion

Boucher first fixed the lumbosacral joint through the pedicle of the vertebra with screws in 1959 and achieved satisfactory results (9). Since then, transpedicular internal fixation has been widely used in clinical and has become the most commonly used fixation method in posterior spinal surgery. Lavaste et al. confirmed the “grip” effect of pedicle by studying the biomechanical properties of pedicle, so pedicle screw into the vertebral body through pedicle can control the “three-column” composite structure of spine and achieve good three-dimensional fixation, And the holding force of pedicle screw is related to the diameter of screw (10). Ke, U.D. proved that there was a positive correlation between pedicle screw holding force and screw diameter, that is, the holding force increased with the increase of screw diameter (11). Experiments made by Kwok et al. showed that the pullout strength of 6.5 mm pedicle screws was 47% higher than that of 5.5 mm screws (12, 13). Polly et al. thought that increasing the diameter of screw for 2 mm in revision surgery is the most ideal method for pedicle screw revision (14). Increasing l mm in diameter and 5–10 mm in length is also a areliable means, so relatively thick screws should be placed as much as possible to achieve sufficient holding force during screw placement.

Thinner pedicles are often encountered in patients. A study of people in Malaysians made by Liau pointed out that T4 was the most common site where the diameter of pedicle was less than 5.5 mm, followed by T5, T6, T7, T8 and T9. The proportion of patients whose diameter of T4-T7 less than 4.5 mm accounted for a large proportion (15). Ru, DuXin et al. studied the shape, internal longitudinal diameter and internal transverse diameter of T11-L5 pedicle in spinal cadaver specimens. The results showed that the internal longitudinal diameter of thoracolumbar vertebrae was larger than 9 mm, while the internal transverse diameter less than 4 mm accounted for 2.6%, 30%, and the reported minimum internal transverse diameter was 2 mm (16). There are a large number of small pedicle diameters in patients with scoliosis, and the pedicle diameter on the concave side of the thoracic vertebra is significantly smaller than that on the convex side (2, 17). At present, the diameter of conventional screw used in clinical operation is 4.0 mm–6.0 mm, so there is a risk of pedicle rupture when conventional screw is placed in the thinner pedicle whose internal transverse diameter is smaller than 4.0 mm, which may lead to decreased screw holding force, nerve injury and other related complications. Therefore, it is very important to explore how to safely place relatively thick pedicle screws in the thinner pedicle.

Wei, X et al. confirmed the expansibility of pedicle diameter through related studies on cadaveric specimens (18); Cahill et al. did experiments on a cadaver of a prepubertal boy and found that the internal diameter of the pedicle could be increased by 74% and the outer diameter by 24% (6); The biomechanical study made by Ke, U.D. et al. confirmed that the holding force of the pedicle screw with the same diameter has no significant difference between the pedicle expansion deformation group and the non-expansion deformation group. There was no significant decrease in the holding force even when the pedicle cortex was cut out by the screw thread (11). Therefore, the insertion of the screw after the pedicle is expanded would not weaken the holding force. The situation that the screw thread cuts out the pedicle cortex and affects the holding force due to thinning can also be ignored accordingly.

The mechanical mechanisms of the expansion of the inner diameter of the pedicle has been revealed by Wei, X et al. (18) that the lateral elastic characteristics of human bone are approximately isotropic, as described by Young's modulus and Poisson's ratio. The Young's modulus determines the elasticity of the material. The harder the material is, the greater the maximum modulus is. The longitudinal Young's modulus of the bone is 1.5 times that of the transverse Young's modulus. therefore, the transverse elasticity of the bone is larger than that of the longitudinal, that is, the transverse strain of the bone is greater than the longitudinal strain under the same stress, so even if the transverse and longitudinal diameter of the pedicle is equal, the effect of screw implantation on the transverse diameter is greater than that on the longitudinal diameter. Poisson's ratio refers to the expansion or shrinkage of the material when it is stretched or extruded. The higher the Poisson's ratio, the more significant the expansion after tension. The Poisson's ratio of compact bone is approximately 0.6, much higher than that of metal. Therefore, when trying to expand the pedicle with metal instruments, the mutual pressure between the instrument and the bone will make the pedicle expand and achieve the expansion of the inner diameter.

Some scholars have studied the artificial enlargement of the internal diameter of the pedicle. Yazici and others have used stainless steel cannula-like distraction instruments to dilate the porcine pedicle. The results showed that the pedicle of the spine can be continuously expanded through an increased size of the expander (19). However, the force of this expansion is explosive, which has a strong influence on the peripheral wall of the pedicle and has a greater risk of injury to the pedicle. On the other hand, the gradual expansion of GEPT emphasizes creep, reduces the risk of pedicle “bursting”, and has a gentler effect on the inner wall of pedicle.

The central position of pedicle should be chosen as far as possible in the application of GEPT, so that the pedicle tends to expand uniformly. If the point of entry is lateral or medial, the degree of expansion may vary due to the difference in the distribution of cancellous bone in the pedicle. Misenhhim et al. have shown that when screws are placed into pedicles with smaller internal diameter, one of the following three situations will occur: pedicle dilatation, pedicle cutting damage or pedicle fracture (20). Because in the process of using the screw, the thread may cut into the bone at these points and affect the cross-sectional integrity of the pedicle. The screw-like head end of GEPT is selected as a blunt and smooth thread, which avoids a greater degree of sharp cutting of the inner wall of the pedicle and is a safer choice. Theoretically, the thread-like injury of the inner wall of the pedicle caused by proper blunt cutting plays a positive role in the maintenance of screw holding force with the healing and reconstruction of the pedicle. This phenomenon is likely to be observed at the microscopic level. Further exploration will be carried out in the next step of research.

In this study, the purpose of placing thicker screws in the thinner pedicle was achieved by expanding the internal diameter of the pedicle. For safety reasons, excessive expansion of the pedicle was not attempted, and the pedicle in this study came from patients with adolescent idiopathic scoliosis. The patients are younger, the pedicle expansion rate may be higher than that of the adult pedicle, and the bone mineral density is generally better than that of the elderly. Therefore, in the future research, we will make an in-depth study on the expansion rate of pedicles of different ages and different parts.

## 5 Conclusion

Our technique shows that in AIS patients, the thinner pedicle with a diameter range of 2.5 mm to 4.5 mm can be safely inserted with relatively thicker pedicle screws after gradually expanding pedicle technology.

## Data Availability

The original contributions presented in the study are included in the article/Supplementary Material, further inquiries can be directed to the corresponding authors.
